# Draft Genome Sequence of a Virulent *Pectobacterium carotovorum* subsp. *brasiliense* Isolate Causing Soft Rot of Cucumber

**DOI:** 10.1128/genomeA.01530-15

**Published:** 2016-01-07

**Authors:** Edward M. Onkendi, Aadi Moolam Ramesh, Stanford Kwenda, Sanushka Naidoo, Lucy Moleleki

**Affiliations:** a Forestry and Agricultural Biotechnology Institute (FABI), Genomics Research Institute (GRI), Pretoria, South Africa; b Department of Microbiology and Plant Pathology, University of Pretoria, Pretoria, South Africa; c Department of Genetics, University of Pretoria, Pretoria, South Africa

## Abstract

*Pectobacterium carotovorum* subsp. *brasiliense* causes soft rot and blackleg diseases on potatoes, ornamentals, and other crops of economic importance. Here, we report a draft genome sequence of a highly virulent *P. carotovorum* subsp. *brasiliense* strain, PcbHPI01, isolated from a cucumber in South Africa.

## GENOME ANNOUNCEMENT

*Pectobacterium carotovorum* subsp. *brasiliense* causes considerable losses in potatoes and vegetables due to blackleg and soft rot diseases. *P. carotovorum* subsp. *brasiliense* was first reported from a potato in Brazil (2004) as a virulent and highly aggressive phytopathogen causing soft rot and blackleg in potatoes (1). To date, *P. carotovorum* subsp. *brasiliense* is a global problem with reports from regions, such as the United States, Israel, South Africa, Canada, New Zealand, South Korea, the Netherlands, and Kenya (2–8). We recently isolated a *P. carotovorum* subsp. *brasiliense* strain from cucumber plants and observed that this isolate (PcbHPI01) is highly virulent and more aggressive than the type strain Pcb1692 when inoculated into both cucumber and potato tubers. The high virulence levels in the cucumber isolate motivated us to sequence its genome. Given that this isolate is from a host other than potato, we hypothesize that comparative genomics with other soft rot *Enterobacteriaceae* strains, most of which were isolated from potatoes, will provide more insight into factors that might be contributing to the high virulence, as well as factors that may determine host specificity. Hence, we report here a draft genome sequence of a *P. carotovorum* subsp. *brasiliense* strain PcbHPI01, isolated from a cucumber in South Africa.

High-quality genomic DNA was extracted from pure cultures using the MasterPure DNA purification kit (Epicentre, WI, USA) and submitted for sequencing. Genome sequencing was done by Illumina HiSeq using High Output version 4 (Fasteris, Switzerland), with paired-end reads of 1 × 125 bp. The 16,010,326 raw reads were checked for quality using FASTQC (http://www.bioinformatics.babraham.ac.uk/projects/fastqc/) and trimmed using Trimmomatic version 0.33 to give 15,286,036 reads with an estimated coverage of 386×. The reads were assembled *de novo* based on the de Bruijn graph algorithm implemented in SPAdes assembler version 3.6. The quality-checked genome sequence was then annotated using the Glimmer option of RAST (9). The draft genome consists of 55 contigs of >500 bp and an *N*_50_ of 404,385 bp. The estimated genome size is 4.8 Mb, with a G+C content of 52.12%. Furthermore, the PcbHPI01 genome has a total of 4,567 predicted features, including 4,488 protein-coding sequences (CDSs), 7 rRNAs, and 72 tRNAs. Of the 4,488 CDSs, 242 putatively code for virulence.

### Nucleotide sequence accession numbers.

The whole-genome shotgun project has been deposited at DDBJ/EMBL/GenBank under the accession number LKKQ00000000. The version described in this paper is version LKKQ01000000.
